# The association between tinnitus and the risk of ischemic cerebrovascular disease in young and middle-aged patients: A secondary case-control analysis of a nationwide, population-based health claims database

**DOI:** 10.1371/journal.pone.0187474

**Published:** 2017-11-02

**Authors:** Yung-Sung Huang, Malcolm Koo, Jin-Cherng Chen, Juen-Haur Hwang

**Affiliations:** 1 Department of Neurology, Dalin Tzu Chi Hospital, Buddhist Tzu Chi Medical Foundation, Dalin, Chiayi, Taiwan; 2 Department of Medical Research, Dalin Tzu Chi Hospital, Buddhist Tzu Chi Medical Foundation, Dalin, Chiayi, Taiwan; 3 Dalla Lana School of Public Health, University of Toronto, Toronto, Ontario, Canada; 4 Department of Neurosurgery, Dalin Tzu Chi Hospital, Buddhist Tzu Chi Medical Foundation, Dalin, Chiayi, Taiwan; 5 School of Medicine, Tzu Chi University, Hualien City, Taiwan; 6 Department of Otolaryngology, Dalin Tzu Chi Hospital, Buddhist Tzu Chi Medical Foundation, Dalin, Chiayi, Taiwan; University of Regensburg, GERMANY

## Abstract

**Background:**

Tinnitus and ischemic cerebrovascular disease (ICVD) may share common pathophysiologic mechanisms. Nevertheless, no studies have investigated whether tinnitus is associated with a higher risk of ICVD. The aim of this study was to evaluate the risk of ICVD among young and middle-aged patients with tinnitus.

**Methods:**

Using the Taiwan’s National Health Insurance Research Database, we identified 3,474 patients 20–45 years old with incident ICVD diagnosed between January 1, 2000 and December 31, 2010 and 17,370 controls, frequency matched on age interval, sex, and year of the index date. Risk of ICVD associated with tinnitus was assessed using multiple logistic regression analyses.

**Results:**

Tinnitus was significantly associated with a higher risk of incident ICVD among young and middle-aged patients (adjusted odds ratio [OR] 1.66, 95% confidence interval [CI] 1.34–2.04), adjusting for sex, age, and comorbidities. In addition, sex-stratified analysis showed that the associations were significant in both male (adjusted OR 1.55, 95% CI 1.16–2.07) and female patients (adjusted OR 1.77, 95% CI 1.30–2.41). Furthermore, tinnitus was significantly associated with a higher risk of ICVD in the 20.0–29.9 years (adjusted OR 4.11, 95% CI 1.98–8.52) and 30.0–39.9 years (adjusted OR 2.19, 95% CI 1.57–3.05) age groups, but not in the 40.0–45.0 years age group.

**Conclusions:**

Tinnitus could be a novel risk factor or clinical indicator for young ischemic stroke, and further investigations are warranted.

## Introduction

Tinnitus is a common medical symptom that can be defined as the conscious perception of an auditory non-speech sensation, such as hissing, sizzling, and ringing, in the absence of a corresponding external sound. Epidemiological studies performed in Western countries have found the prevalence of tinnitus to be approximately 10% of the adult population, regardless of sex [1]. A recent cross-sectional survey in South Korea showed that the prevalence of tinnitus was 20.7%, and the proportions with no associated discomfort, moderate annoyance, and severe annoyance were 69.2%, 27.9%, and 3.0%, respectively [2].

Tinnitus can occur in association with a number of otological diseases, including otosclerosis, Ménière’s disease, and vestibular schwannoma, as well as other metabolic abnormalities and psychiatric disturbances [3,4]. Specifically, sensorineural hearing loss, obesity, smoking, alcohol consumption, previous head injuries, hypertension, sleep disturbances, and certain medications are a few of the possible risk factors for the sensorineural-type tinnitus [5]. In addition, tinnitus was found to be associated with the use of diuretics and low systolic blood pressure in patients receiving antihypertensive therapy [6]. Patients with severe cases of tinnitus can suffer from sleep problems [7], anxiety [8], and depressive symptoms [9]. Nevertheless, to the best of our knowledge, no studies have yet investigated whether tinnitus is associated with a higher risk of ischemic cerebrovascular disease (ICVD).

Sex, hypertension, diabetes, dyslipidemia, smoking, age and metabolic syndrome are major vascular risk factors for intracranial or extracranial atherosclerotic stenosis in Asian population [10]. Specifically, strokes in young adults are reported as being uncommon, comprising 10%–15% of all stroke patients. Young stroke patients have an increased mortality compared with the general population. The prevalence of standard modifiable vascular risk factors in young stroke patients is also different from that of older patients, in which dyslipidemia, smoking, and hypertension are highly prevalent in the young stroke population [11]. In addition, migraine with aura was found to be an independent risk factor for ischemic stroke in young population and in women [12].

It is plausible that tinnitus and ICVD may share common pathophysiologic mechanisms such as arterial stiffening. Arterial stiffness can lead to impaired cochlear microcirculation. A higher common carotid artery stiffness index was found to be significantly associated with the formation and severity of tinnitus [13]. Arterial stiffness is also associated with a higher incidence of stroke [14]. Therefore, the aim of this study is to evaluate the risk of incident ICVD among young and middle-aged patients with tinnitus, using a nationwide, population-based health claims research database.

## Methods

### Data source

The data source of this retrospective secondary case-control study was based on the Longitudinal Health Insurance Database 2000 (LHID 2000), which is a subset of the National Health Insurance Research Database (NHIRD). The LHID 2000 dataset included the claim records of approximately one million randomly sampled patients from the NHIRD, which represents about 5% of the total population in Taiwan [15].

The study was approved by the institutional review board of the Dalin Tzu Chi Hospital, Buddhist Tzu Chi Medical Foundation, Taiwan (No. B10202021). Since the NHIRD files contain only de-identified secondary data, the review board waived the requirement for obtaining informed consent from the patients.

### Identification of cases and controls

**Fig 1:**
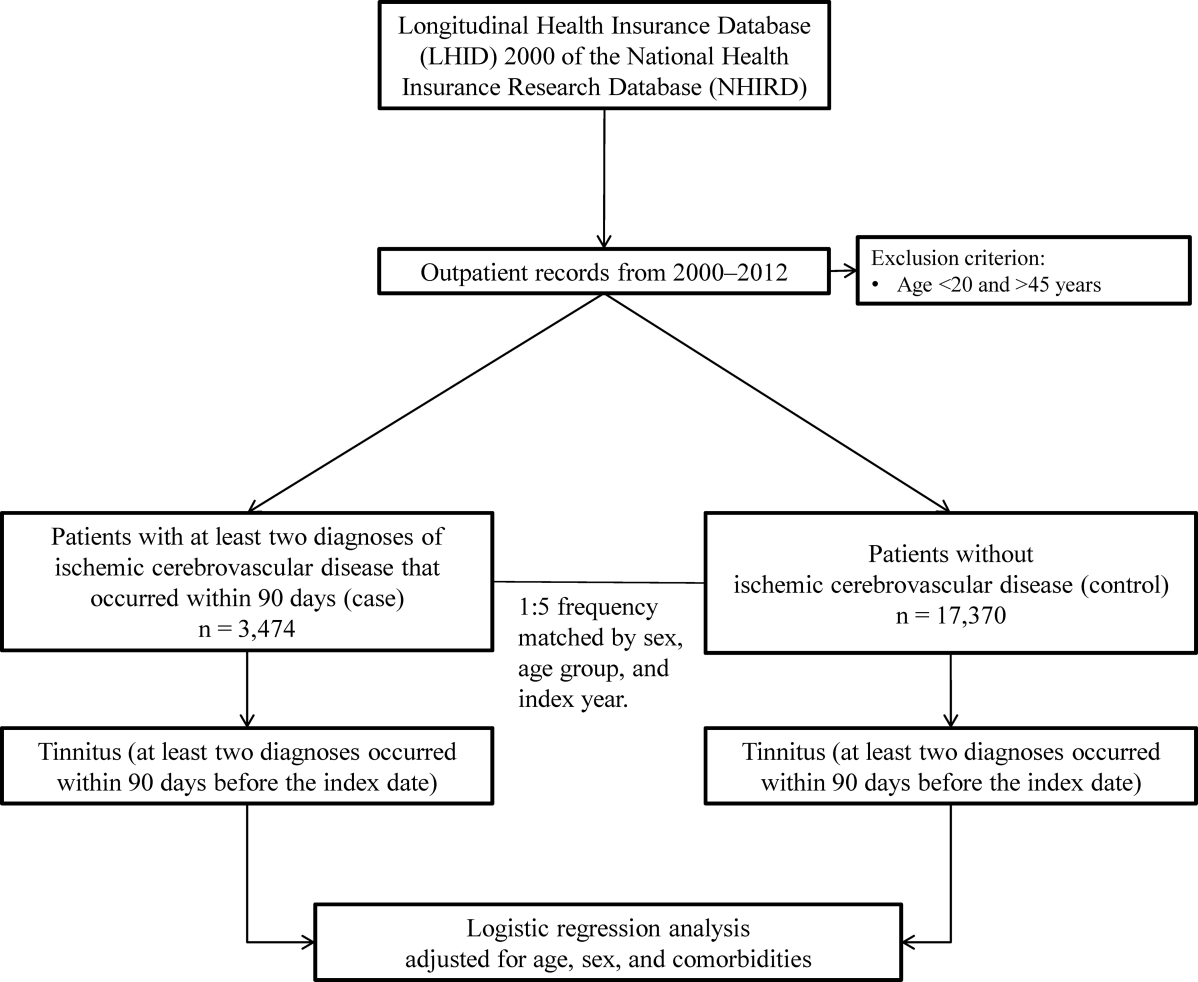
Study flowchart.

Patients were defined as incident ICVD cases if they had at least two newly diagnoses of specific International Classification of Diseases, Ninth Revision, Clinical Modification (ICD-9-CM) codes for ICVD (S1 Table) in the period between 2000 and 2012. The two diagnoses had to occur within a period of 90 days. Controls were patients without ICVD, frequency matched by sex, age group, and index year, with a control to case ratio of 5:1 (Fig 1). The exclusion criteria were (1) patients who were younger than 20.0 years or older than 45.0 years of age and (2) patients with ICVD in 1996–1999.

### Identification of tinnitus and comorbidity

The main risk factor of interest in this study was tinnitus, which was defined as at least two outpatient diagnoses of an ICD-9-CM code of 388.3 that occurred within a period of 90 days in the period between 2000 and 2012. In addition, the diagnoses of tinnitus had to occur before the index date. The comorbidities evaluated in this study included atrial fibrillation, benign brain tumor, coronary artery disease or myocardial infarction, chronic hepatitis, chronic kidney disease, chronic obstructive pulmonary disease, concussion or head trauma, conductive hearing loss, diabetes mellitus, hyperlipidemia, hypertension, liver cirrhosis, malignant brain tumor, Ménière's disease, obesity, obstructive sleep apnea, Parkinson’s disease, peripheral arterial occlusive disease, sensorineural hearing, sleep disturbance, sudden sensorineural hearing loss, vertigo, and vestibular schwannoma. These comorbid conditions were defined based on specific ICD-9-CM codes (S1 Table) and if they were diagnosed at least twice within a period of 90 days. In addition, the diagnoses of these comorbid conditions had to occur prior to the index date.

### Statistical analysis

Statistical analyses were performed using IBM SPSS software package, version 24.0 (IBM Corp, Armonk, NY, USA). A *P* value of < 0.05 was considered significant. Distributions of age, sex, and comorbidities between cases and controls were evaluated using Chi-squared test or Fisher’s exact test, as appropriate. Unconditional multiple logistic regression analyses were used to evaluate the risks of ICVD associated with tinnitus with and without adjusting for age, sex, and comorbidities, respectively. Adjusted models with significant covariates were built based on backward selection using likelihood ratio test. Moreover, sub-group analyses were conducted with stratification by sex and age groups.

## Results

Table 1 shows the distribution of age group, sex, and comorbidities for the 3,474 patients with ICVD and the 17,370 controls. There were no significant differences in the distribution of age interval and sex as a result of the frequency matching on these two variables.

**Table 1:** Characteristics of the ischemic cerebrovascular disease cases and controls (N = 20,844).

Variable	*n* (%)		*P* Value
	Case 3,474 (16.7)	Control 17,370 (83.3)	
Age Group (years)			> 0.999
20.0–29.9	517 (14.9)	2,585 (14.9)	
30.0–39.9	1,413 (40.7)	7,065 (40.7)	
40.0–45.0	1,544 (44.4)	7,720 (44.4)	
Sex			> 0.999
male	1,973 (56.8)	9,865 (56.8)	
female	1,501 (43.2)	7,505 (43.2)	
Comorbidity			
atrial fibrillation	5 (0.1)	15 (0.1)	0.361
benign brain tumor	30 (0.9)	25 (0.1)	< 0.001
chronic kidney disease	53 (1.5)	114 (0.7)	< 0.001
concussion or head trauma	294 (8.5)	564 (3.2)	< 0.001
conductive hearing loss	2 (0.1)	3 (0.02)	0.196
chronic obstructive pulmonary disease	160 (4.6)	577 (3.3)	< 0.001
coronary artery disease or myocardial infarction	179 (5.2)	344 (2.0)	< 0.001
diabetes mellitus	296 (8.5)	712 (4.1)	< 0.001
hyperlipidemia	436 (12.6)	1,228 (7.1)	< 0.001
hypertension	781 (22.5)	1,409 (8.1)	< 0.001
liver cirrhosis	165 (4.7)	723 (4.2)	0.118
malignant brain tumor	25 (0.7)	10 (0.1)	< 0.001
Ménière's disease	66 (1.9)	131 (0.8)	< 0.001
obesity	42 (1.2)	148 (0.9)	0.043
obstructive sleep apnea	22 (0.6)	53 (0.3)	0.003
Parkinson’s disease	17 (0.5)	27 (0.2)	< 0.001
peripheral arterial occlusive disease	28 (0.8)	76 (0.4)	0.005
sensorineural hearing loss	48 (1.4)	116 (0.7)	< 0.001
sleep disturbance	535 (15.4)	1,998 (11.5)	< 0.001
sudden deafness	10 (0.3)	23 (0.1)	0.035
vertigo	168 (4.8)	271 (1.6)	< 0.001
vestibular schwannoma	2 (0.1)	7 (0.04)	0.651

Results for the multiple logistic regression analyses with and without stratification by sex or by age group are shown in Table 2. After adjusting for sex, age, and comorbidities, tinnitus was significantly associated with a higher risk of ICVD among our sample of young and middle-aged patients (adjusted odds ratio [OR] 1.66, 95% confidence interval [CI] 1.34–2.04). The full model is presented in S2 Table. Similar results were obtained when the analysis was stratified by sex. The association was significant in both male (adjusted OR 1.56, 95% CI 1.17–2.08) and female patients (adjusted OR 1.77, 95% CI 1.30–2.41). The two full models are presented in S3 Table. When the data were stratified by age group, tinnitus was significantly associated with a higher risk of ICVD in the 20.0–29.9 years (adjusted OR 4.09, 95% CI 1.97–8.49) and 30.0–39.9 years (adjusted OR 2.22, 95% CI 1.59–3.08) age groups with a larger magnitude of OR in the younger age group. However, for the 40.0–45.0 years age group, the association was not significant (adjusted OR 1.20, 95% CI 0.77–1.48). The three full models are presented in S4 Table.

**Table 2:** Multiple logistic regression analysis of the risk of ischemic cerebrovascular disease in patients with tinnitus, with stratification by sex or by age group (N = 20,844).

		n (%)		Adjusted Odds Ratio (95% CI)
		Case 3,474	Control 17,370	
Tinnitus, all patients		143 (4.1)	360 (2.1)	1.66 (1.34–2.04)
Tinnitus, stratified by sex				
	Male	74 (3.8)	201 (2.0)	1.56 (1.17–2.08)
	Female	69 (4.6)	159 (2.1)	1.77 (1.30–2.41)
Tinnitus, stratified by age group (years)				
	20.0–29.9	15 (2.9)	17 (0.7)	4.09 (1.97–8.49)
	30.0–39.9	68 (4.8)	119 (1.7)	2.22 (1.59–3.08)
	40.0–45.0	60 (3.9)	224 (2.9)	1.20 (0.89–1.63)

CI, confidence interval.

Variables evaluated in the multiple logistic regression model using backward likelihood ratio method included age, sex (except in the analysis with stratification by sex), and comorbidities (atrial fibrillation, benign brain tumor, chronic kidney disease, chronic obstructive pulmonary disease, concussion or head trauma, conductive hearing loss, coronary artery disease or myocardial infarction, diabetes mellitus, hyperlipidemia, hypertension, liver cirrhosis, malignant brain tumor, Ménière's disease, obesity, obstructive sleep apnea, Parkinson’s disease, peripheral arterial occlusive disease, sensorineural hearing impairment, sleep disturbance, sudden deafness, vertigo, and vestibular schwannoma). The variables remained in the final models are presented in S2–S4 Tables.

## Discussion

This secondary case-control study using the nationwide, population-based health claims data of the Taiwan’s NHIRD showed that tinnitus was significantly associated with a higher risk of ICVD among young and middle-aged adults, particularly in those individuals under 40 years of age. These findings suggested that tinnitus might be a novel risk factor or an indicator for young ICVD.

Several mechanisms for stroke have been described, including artery-to-artery embolism (59.7%), local branch occlusion (14.9%), in situ thrombo-occlusion (13.7%), hemodynamic impairment (0.9%), and mixed (10.8%) [16]. The prevalence of risk factors and stroke mechanisms differed between intracranial atherosclerotic stenosis (ICAS) and extracranial atherosclerotic stenosis (ECAS), and between anterior and posterior circulation atherosclerosis. Female sex and metabolic syndrome were more closely associated with ICAS in posterior circulation strokes, while age, smoking, and dyslipidemia were more associated with ECAS [10,16,17].

The most common risk factors for young ischemic stroke are dyslipidemia, smoking, hypertension, patent foramen ovale, diabetes mellitus, and a family history of stroke [18,19]. Similarly, a study of 264 patients in Taiwan also reported that the four most common risk factors for young ischemic stroke were hyperlipidemia, smoking, hypertension, and a family history of stroke [20]. Other rarer causes of young stroke included severe iron-deficiency anemia with or without reactive thrombocytosis [18], Moyamoya syndrome [21], and Takayasu arteritis [22]. In addition, intracranial stenosis was reported to be more common than extracranial stenosis in both the carotid and vertebrobasilar systems of young ischemic stroke in Taiwan [20]. However, other studies indicated that cervical artery dissections are among the most common causes of ischemic stroke in young and middle-aged adults [23,24].

Tinnitus is often associated with sensorineural hearing impairment and arterial hypertension [25]. The Blue Mountains Hearing Study reported a significantly higher risk of reporting previous stroke in patients with moderate to severe hearing loss (OR 2.04, 95% CI 1.20–3.49). However, moderate to severe hearing loss was not associated with a higher incident stroke after five-year follow-up (OR 1.14, 95% CI 0.59–2.23) [26]. Previous studies have reported a strong association between tinnitus and young stroke. For example, pulsatile tinnitus, ischemic stroke, migraine, Horner's syndrome, and subarachnoid hemorrhage were found in patients with internal carotid artery agenesis [27]. A case report of a young male patient who had a progressive carotid artery dissection was reported to have left sided tinnitus and ipsilateral head and neck pain [28]. Clinical manifestations of spontaneous cervicocephalic artery dissection included ischemic stroke, transient ischemic attack, headache, neck pain, Horner syndrome, pulsatile tinnitus, and dysgeusia [29]. Tinnitus was also one of the symptoms of superior cerebellar artery infarction [30] and anterior inferior cerebellar artery infarction [31]. But cerebral infarctions of the basal ganglia, thalamus and pons were inversely associated with tinnitus. Furthermore, brain atrophy, ventricular dilatation, and white matter lesions had no significant effects on the prevalence of tinnitus [32].

The association between tinnitus and stroke could be explained by several shared pathophysiological mechanisms, such as arterial stiffening [13,14], hypoxia [33], oxidative stress [34,35], neural inflammation [36–39], poor sleep [5], and increased sympathetic activity [40]. Thus, tinnitus could precede the occurrence of stroke not only as an intermediate role in the association between vascular disease and stroke, but also as an independent risk factor for stroke. Further research is warranted to elucidate the underlying mechanisms.

To our knowledge, this study is the first to report an increase risk of ICVD in young patients with tinnitus. Nevertheless, a few limitations should be taken into account when interpreting the findings. First, there is a lack of information on the severity of tinnitus, which is a limitation common to all studies based on analyses of the NHIRD administrative database. Second, the character of tinnitus (for example, non-pulsatile or pulsatile and intermittent or persistent) was unknown and therefore, the impact of different types of tinnitus on the risk of young stroke could not be further examined. Third, the influence from body mass index and smoking habits could only be adjusted using a diagnosis of obesity and chronic obstructive pulmonary disease, respectively. Nevertheless, the association between ICVD and tinnitus observed in this study should not be a result of confounding by smoking because similar associations were observed in both men and women despite that the prevelance of smoking were drastically different between the two sexes. In Taiwan, the prevalence of smoking in men and women aged 25 to 39 years was approximately 38% and 7%, respectively [41].

In conclusion, this secondary case-control study of a nationwide, population-based health claims database showed that tinnitus was associated with a significantly higher risk of ICVD, particularly among adults under 40 years of age. Tinnitus could be a novel risk factor or clinical indicator for young ischemic stroke, and further investigations are warranted.

## Supporting information

S1 Table International Classification of Diseases, Ninth Revision, Clinical Modification (ICD-9-CM) diagnosis codes for ischemic cerebrovascular disease, tinnitus, and comorbidities. (DOC)

S2 Table Multiple logistic regression analysis of the risk of ischemic cerebrovascular disease in patients with tinnitus. (DOC)

S3 Table Multiple logistic regression analysis of the risk of ischemic cerebrovascular disease in male and female patients with tinnitus. (DOC)

S4 Table Multiple logistic regression analysis of the risk of ischemic cerebrovascular disease in patients with tinnitus, stratified by age group. (DOC)
