# Supplementary material for: The association between tinnitus and the risk of ischemic cerebrovascular disease in young and middle-aged patients: A secondary case-control analysis of a nationwide, population-based health claims database
Source: PLoS One. 2017 Nov 2;12(11):e0187474. doi: 10.1371/journal.pone.0187474 (PMC5667787; doi:10.1371/journal.pone.0187474)
Supplement: S1 Table — (DOC) [file pone.0187474.s001.doc]

Supplement S1 Table. International Classification of Diseases, Ninth Revision, Clinical Modification (ICD-9-CM) diagnosis codes for ischemic cerebrovascular disease, tinnitus, and comorbidities.

| **Code** | **Diagnosis** |
| --- | --- |
| **Codes for ischemic cerebrovascular disease** | |
| 433.01 | Occlusion and stenosis of basilar artery with cerebral infarction |
| 433.10 | Occlusion and stenosis of carotid artery without mention of cerebral infarction |
| 433.11 | Occlusion and stenosis of carotid artery with cerebral infarction |
| 433.21 | Occlusion and stenosis of vertebral artery with cerebral infarction |
| 433.90 | Occlusion and stenosis of unspecified precerebral artery without mention of cerebral infarction |
| 434.xx | Occlusion of cerebral arteries |
| 435.1 | Vertebral artery syndrome |
| 435.3 | Vertebrobasilar artery syndrome |
| 435.8 | Other specified transient cerebral ischemias |
| 435.9 | Unspecified transient cerebral ischemia |
| 436 | Acute, but ill-defined, cerebrovascular disease |
| 437.0 | Cerebral atherosclerosis |
| 437.8 | Other ill-defined cerebrovascular disease |
| 437.9 | Unspecified cerebrovascular disease |
| 438.89 | Other late effects of cerebrovascular disease |
| 438.9 | Unspecified late effects of cerebrovascular disease |
| **Code for tinnitus** | |
| 388.3 | Tinnitus |
| **Codes for comorbidities** | |
| 427.31 | Atrial fibrillation |
| 225.x | Benign brain tumor |
| 414, 410, or 429 | Coronary artery disease or myocardial infarction |
| 070 or 571.4 | Chronic hepatitis |
| 585–586 | Chronic kidney disease |
| 491, 492, or 496 | Chronic obstructive pulmonary disease |
| 800–804, 850–854, 8730, 8731, or 310.2 | Concussion or head trauma |
| 389.0x | Conductive hearing loss |
| 250 | Diabetes mellitus |
| 272 | Hyperlipidemia |
| 401–405 | Hypertension |
| 571, 571.2, 571.5, or 571.6 | Liver cirrhosis |
| 191.x or 192.x | Malignant brain tumor |
| 386.0 | Ménière's disease |
| 278.0, 278.00, 278.01, or 278.02 | Obesity |
| 780.51, 780.53, or 780.57 | Obstructive sleep apnea |
| 332 or 094.82 | Parkinson’s disease |
| 443 | Peripheral arterial occlusive disease |
| 388.12, 388.40, 389.1, 389.2, 389.7, 389.8, or 389.9 | Sensorineural hearing impairment |
| 780.50, 780.52, or 307.4 | Sleep disturbance |
| 388.2 | Sudden sensorineural hearing loss |
| 386.1, 386.2, 386.5, 386.8, or 386.9 | Vertigo |
| 225.1 | Vestibular schwannoma |
