# Supplementary material for: The association between tinnitus and the risk of ischemic cerebrovascular disease in young and middle-aged patients: A secondary case-control analysis of a nationwide, population-based health claims database
Source: PLoS One. 2017 Nov 2;12(11):e0187474. doi: 10.1371/journal.pone.0187474 (PMC5667787; doi:10.1371/journal.pone.0187474)
Supplement: S2 Table — (DOC) [file pone.0187474.s002.doc]

Supplement S2 Table. Multiple logistic regression analysis of the risk of ischemic cerebrovascular disease in patients with tinnitus.

| Variable | odds ratio (95% CI) | *P* |
| --- | --- | --- |
| tinnitus | 1.66 (1.34–2.04) | < 0.001 |
| sex (reference category: male) | 1.11 (1.03–1.20) | 0.006 |
| benign brain tumor | 3.53 (1.93–6.45) | < 0.001 |
| concussion or head trauma | 2.60 (2.24–3.03) | < 0.001 |
| coronary artery disease or myocardial infarction | 1.55 (1.27–1.89) | < 0.001 |
| diabetes mellitus | 1.45 (1.24–1.68) | < 0.001 |
| hypertension | 2.89 (2.60–3.20) | < 0.001 |
| malignant brain tumor | 9.02 (4.10–19.88) | < 0.001 |
| Ménière's disease | 1.50 (1.08–2.07) | 0.014 |
| Parkinson’s disease | 2.63 (1.38–5.00) | 0.003 |
| vertigo | 2.24 (1.81–2.76) | < 0.001 |

CI: confidence interval
