# Supplementary material for: The association between tinnitus and the risk of ischemic cerebrovascular disease in young and middle-aged patients: A secondary case-control analysis of a nationwide, population-based health claims database
Source: PLoS One. 2017 Nov 2;12(11):e0187474. doi: 10.1371/journal.pone.0187474 (PMC5667787; doi:10.1371/journal.pone.0187474)
Supplement: S3 Table — (DOC) [file pone.0187474.s003.doc]

Supplement S3 Table. Multiple logistic regression analysis of the risk of ischemic cerebrovascular disease in male and female patients with tinnitus.

| Sex | Variable | odds ratio (95% CI) | *P* |
| --- | --- | --- | --- |
| **Male** |  |  |  |
|  | tinnitus | 1.56 (1.17–2.08) | 0.003 |
|  | concussion or head trauma | 2.49 (2.07–3.01) | < 0.001 |
|  | coronary artery disease or myocardial infarction | 1.49 (1.16–1.92) | 0.002 |
|  | diabetes mellitus | 1.52 (1.26–1.82) | < 0.001 |
|  | hypertension | 2.88 (2.53–3.27) | < 0.001 |
|  | malignant brain tumor | 7.85 (3.31–18.59) | < 0.001 |
|  | Parkinson’s disease | 2.85 (1.35–6.01) | 0.006 |
|  | sleep apnea | 1.80 (1.02–3.16) | 0.042 |
|  | vertigo | 2.60 (1.91–3.52) | < 0.001 |
| **Female** |  |  |  |
|  | tinnitus | 1.77 (1.30–2.41) | < 0.001 |
|  | benign brain tumor | 6.14 (2.74–13.75) | < 0.001 |
|  | concussion or head trauma | 2.86 (2.21–3.70) | < 0.001 |
|  | coronary artery disease or myocardial infarction | 1.68 (1.21–2.34) | 0.002 |
|  | hyperlipidemia | 1.32 (1.04–1.68) | 0.021 |
|  | hypertension | 2.88 (2.40–3.45) | < 0.001 |
|  | malignant brain tumor | 40.43 (4.93–331.52) | 0.001 |
|  | Ménière's disease | 1.82 (1.19–2.77) | 0.005 |
|  | vertigo | 1.96 (1.46–2.63) | < 0.001 |

CI: confidence interval
