# Supplementary material for: The association between tinnitus and the risk of ischemic cerebrovascular disease in young and middle-aged patients: A secondary case-control analysis of a nationwide, population-based health claims database
Source: PLoS One. 2017 Nov 2;12(11):e0187474. doi: 10.1371/journal.pone.0187474 (PMC5667787; doi:10.1371/journal.pone.0187474)
Supplement: S4 Table — (DOC) [file pone.0187474.s004.doc]

Supplement S4 Table. Multiple logistic regression analysis of the risk of ischemic cerebrovascular disease in patients with tinnitus, stratified by age group.

| Age group | Variable | odds ratio (95% CI) | *P* |
| --- | --- | --- | --- |
| **20.0–29.9 years** |  |  |  |
|  | tinnitus | 4.09 (1.97–8.49) | < 0.001 |
|  | chronic obstructive pulmonary disease | 2.05 (1.03–4.08) | 0.040 |
|  | concussion or head trauma | 4.32 (2.99–6.24) | < 0.001 |
|  | coronary artery disease or myocardial infarction | 3.39 (1.11–10.39) | 0.033 |
|  | hypertension | 4.32 (2.50–7.48) | < 0.001 |
|  | liver cirrhosis | 2.46 (1.14–5.28) | 0.021 |
|  | malignant brain tumor | 12.49 (1.14–136.75) | 0.039 |
|  | vertigo | 3.33 (1.57–7.08) | 0.002 |
| **30.0–39.9 years** |  |  |  |
|  | tinnitus | 2.22 (1.59–3.08) | < 0.001 |
|  | age (per 1-year increment) | 1.09 (1.07–1.11) | < 0.001 |
|  | sex (reference category: male) | 1.15 (1.02–1.30) | 0.021 |
|  | benign brain tumor | 5.28 (1.96–14.26) | 0.001 |
|  | coronary artery disease or myocardial infarction | 2.15 (1.54–3.00) | < 0.001 |
|  | concussion or head trauma | 2.30 (1.82–2.91) | < 0.001 |
|  | diabetes mellitus | 1.74 (1.35–2.24) | < 0.001 |
|  | hypertension | 3.71 (3.11–4.42) | < 0.001 |
|  | malignant brain tumor | 3.80 (1.09–13.30) | 0.037 |
|  | Ménière's disease | 1.99 (1.16–3.41) | 0.012 |
|  | vertigo | 2.34 (1.66–3.30) | < 0.001 |
| **40.0–45.0 years** |  |  |  |
|  | tinnitus | 1.20 (0.89–1.63) | 0.237 |
|  | age (per 1-year increment) | 1.06 (1.02–1.11) | 0.002 |
|  | benign brain tumor | 3.47 (1.60–7.52) | 0.002 |
|  | concussion or head trauma | 2.31 (1.81–2.95) | < 0.001 |
|  | diabetes mellitus | 1.31 (1.07–1.59) | 0.009 |
|  | hypertension | 2.54 (2.22–2.90) | < 0.001 |
|  | malignant brain tumor | 18.84 (5.18–68.63) | < 0.001 |
|  | vertigo | 2.26 (1.70–3.01) | < 0.001 |

CI: confidence interval
